# Factors associated with heterogeneity in microarray gene expression in peripheral blood mononuclear cells from large pedigrees

**DOI:** 10.1186/s12919-016-0011-3

**Published:** 2016-10-18

**Authors:** Michael Gallaugher, Angelo J. Canty, Andrew D. Paterson

**Affiliations:** 1 Department of Mathematics and Statistics, McMaster University, Hamilton, ON L8S 4 K1 Canada; 2 Genetics and Genome Biology Program, The Hospital for Sick Children Research Institute, Toronto, ON M5G 0A4 Canada; 3 Dalla Lana School of Public Health, University of Toronto, Toronto, ON M5G 0A4 Canada

## Abstract

**Background:**

Genome-wide microarray expression is a rich source of functional genomic data. We examined evidence for differences in expression from peripheral blood mononuclear cells between individuals, examined some of factors that may be responsible and provide recommendations for analysis.

**Methods:**

A total of 643 individuals from 17 large Mexican American pedigrees had microarray gene expression data generated from peripheral blood mononuclear cells. This data has previously been used to map *cis*- and *trans*-expression quantitative trait loci using genome-wide linkage analysis. We estimated both principal components and cell proportions in these data, and tested them for association with clinical factors to provide insight into causes of variation in gene expression between individuals.

**Results:**

We identified that there were highly significant differences in the second principal component of gene expression between pedigrees, with 3 pedigrees being outliers. The estimated cell proportions identified 1 individual who was a gross outlier, as well as pedigrees that differed from others in their estimated proportions of helper and cytotoxic T cells.

**Conclusions:**

These phenomena could be from either pedigree-specific genetic variation, technical artefacts, or clinical factors. Incorporating factors that influence gene expression into genetic analysis, and exclusion of outliers could improve the power of genetic mapping of expression traits.

## Background

Functional genomics studies face many challenges, including defining the cell type(s) of study, and their relative proportions. In the Genetic Analysis Workshop 19 (GAW19) Mexican American family data [1], microarray gene expression data were obtained from 647 individuals after peripheral blood samples were subjected to cell separation using Histopaque® (Sigma Chemical Co.) which separates mononuclear from polynuclear cells [2]. This is expected to remove eosinophils, neutrophils, and basophils, leaving T-, B-, NK-lymphocytes and monocytes. The proportions of T- and B-lymphocytes and monocytes vary between individuals, are heritable, and genome-wide association studies have identified numerous loci for them (eg, Nalls et al. [3]). Gene expression analysis performed on mixtures of cell types can potentially be confounded by heterogeneity of cell types. Similar observations have been made for epigenetic studies using DNA methylation [4].

## Methods

We centered and scaled the gene expression data for each probe to have mean 0 and variance 1. Using a singular value decomposition of the 643 × 20,634 scaled matrix, we found the loadings for the principal components (PCs) and the proportion of total variability accounted for by each (R prcomp). The expression PCs were then used as the response variable to examine the relationship between them and covariates. Specifically, for each of the first 3 PCs we tested for their association with age, gender, medication, blood pressure (BP), hypertension, and smoking status, all measured at visit 1, as well as with pedigree number, one at a time. We also fitted the models for age stratified by gender and, finally, a model with age, gender, and their interaction along with pedigree number. BP measures were missing for 12 individuals at visit 1. Treated systolic blood pressure (SBP) and diastolic blood pressure (DBP) values had 10 and 5 mmHg added to their measured values, respectively, as suggested previously [5]. Statistical significance was defined as *p* < 0.05.

Estimation of the proportion of cytotoxic (CD8+), helper (CD4+) T-, and B-lymphocytes and monocytes in peripheral blood mononuclear cells from each individual was achieved by identifying gene expression signatures for different cell types from HaemAtlas [6] using 4879 probes that overlapped with the GAW19 data, and the quadratic programming algorithm of Gong et al. [7] as implemented in the R package CellMix [8]. Examination of differences in each of the cell proportions between pedigrees was estimated using analysis of variance.

## Results

### Descriptive statistics

Of the 647 individuals with gene expression data, 4 did not appear in the phenotype or pedigree files, leaving 643 for analysis. Of those with expression data, at visit 1, the mean age was 39.6 years (SD = 16.9), with 269 males and 374 females. There were 497 nonsmokers, 133 smokers, and 13 with missing smoking data (coded as a third category in the analysis). A total of 123 had hypertension (SBP >140 mmHg or DBP >90 mmHg or on anti-BP medication), 513 did not, with 7 missing. In addition, 631 individuals had BP measurements (12 missing). Of those with BP data, 559 reported they did not take BP medication, 65 reported taking medications, and 7 were unknown. We only adjusted the 65 individuals who reported taking medications (SBP +10 mmHg, DBP +5 mmHg). The mean unadjusted SBP was 121.9 mmHg (122.9 adjusted), SD = 19.4 (20.9 adjusted). The mean unadjusted DBP was 71.2 mmHg (71.8 adjusted), SD = 9.97 (10.5 adjusted).

### Association of gene expression principal components with covariates

**Fig. 1:**
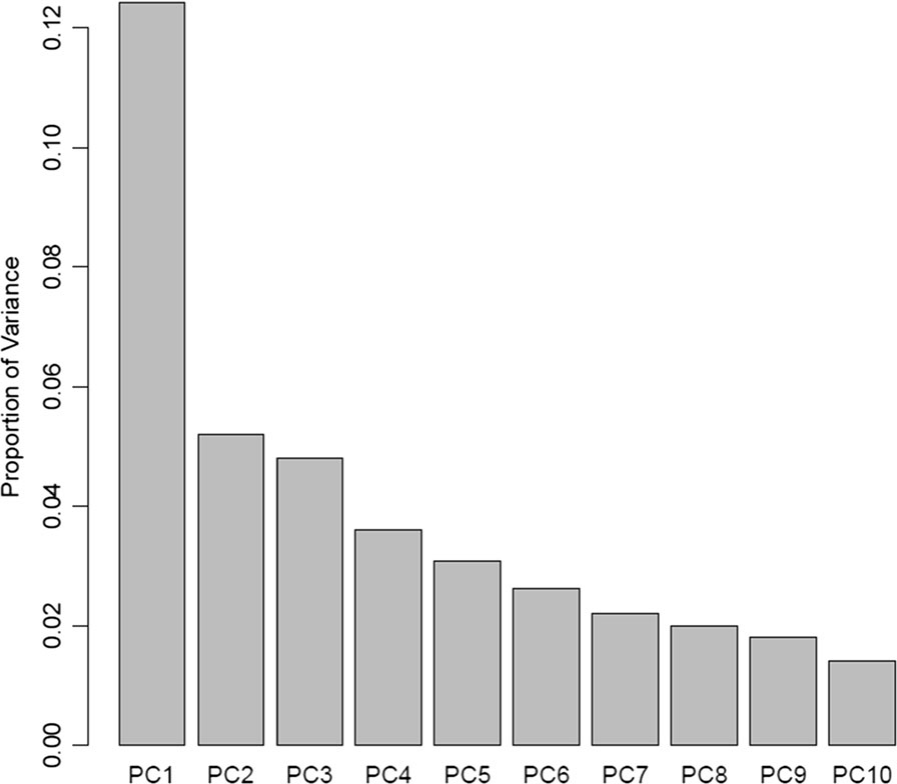
PC analysis of gene expression. Scree plot of the proportion of total variability explained by the first 10 PCs of the expression data

Gene expression data was available for individuals from 17 pedigrees (Table 1). PC analysis identified 26 PCs that account for 50 % of the original variability (Fig. 1). The first 3 PCs account for approximately 12, 5, and 4 %, respectively, of the variance in gene expression with the first 10 PCs accounting for the majority of the variation. For the first 3 PCs, there was only a nominally significant association of PC3 with age (Table 2), but not for PC1 or PC2. Gender was not significantly associated with any of the 3 PCs. There was no significant association of age with first 3 PCs in males (*p* > 0.05), but there was a borderline association within females for PC3, *p* = 0.03. Models including age, gender and their interaction did not identify significant interactions for any of the first 3 PCs. There does appear to be a slight relationship between BP medication and the first and third PCs (Table 2). Medicated individuals had lower values of these 2 PCs. A logistic regression of medication as the response with the first 3 PCs as predictors also showed that the probability of medication decreases as the first (*p* = 0.015) and third (*p* = 0.025) PCs increase, but there is no effect on the probability of medication for the second PC (*p* = 0.74). There was no significant association of SBP with each of the 3 PCs, nominal evidence for association of DBP with PC3. Hypertension status was associated with PC1 (*p* = 0.016, Table 2).

**Table 1:** Association of pedigree number with PC1, PC2, proportions of cytotoxic and helper T cells

Pedigree number	# members	PC1			PC2			Cytotoxic T cells			Helper T cells		
		B	SE	P	B	SE	P	B	SE	P	B	SE	P
2	67	−15.3	6.1	0.013	−6.3	3.6	0.083	−4.6 e-3	5.2 e-3	0.37	7.7e-3	5.1 e-3	0.14
3	44	−10.7	7.5	0.16	−3.9	4.5	0.39	**−1.8 e-2**	**5.9 e-3**	**0.0021**	**2.0e-2**	**5.8 e-3**	**6.6e-4**
4	39	8.7	8.0	0.28	**14.9**	**4.7**	**0.0018**	−1.0 e-2	6.3 e-3	0.12	9.5e-3	6.2 e-3	0.13
5	55	−0.4	6.7	0.94	**−18.4**	**4.0**	**5.6e-6**	−1.6 e-2	5.7 e-3	0.0046	**1.7e-2**	**5.6 e-3**	**0.0022**
6	45	1.6	7.4	0.83	**−14.9**	**4.4**	**8.6e-4**	−1.4 e-2	6.0 e-3	0.019	1.4e-2	6.0 e-3	0.015
8	62	−1.5	6.3	0.81	**33.8**	**3.7**	**<2e-16**	1.3 e-3	5.6 e-3	0.81	−2.5e-3	5.5e-3	0.64
10	49	−6.8	7.1	0.34	−4.8	4.2	0.26	−6.4 e-3	5.6 e-3	0.26	6.5e-3	5.6e-3	0.24
14	30	−10.5	9.1	0.25	2.9	5.4	0.59	−1.6 e-2	6.7 e-3	0.015	1.5e-2	6.6e-3	0.019
15	27	17.1	9.6	0.076	3.0	5.7	0.59	−1.8 e-2	7.0 e-3	0.0086	1.6 e-2	6.9 e-3	0.019
16	38	−4.3	8.1	0.60	−3.6	4.8	0.45	−2.9 e-3	6.1 e-3	0.63	3.3 e-3	6.0 e-3	0.58
17	29	8.6	9.3	0.36	4.2	5.5	0.44	−6.7 e-3	6.7 e-3	0.32	1.3 e-3	6.6 e-3	0.84
20	26	−1.6	9.8	0.86	−7.9	5.8	0.18	7.0 e-4	7.2 e-3	0.92	7.4 e-4	7.1 e-3	0.92
21	30	2.5	9.1	0.78	2.5	5.4	0.63	−1.1 e-2	6.7 e-3	0.084	9.5 e-3	6.6 e-3	0.15
23	28	4.4	9.5	0.64	−13.3	5.6	0.018	1.0 e-3	6.8 e-3	0.88	7.5 e-3	6.7 e-3	0.27
25	22	4.9	10.7	0.65	−4.1	6.3	0.52	5.3 e-3	7.6 e-3	0.48	−3.4 e-4	7.4 e-3	0.96
27	31	24.1	9.0	0.0076	15.0	5.3	0.0051	**−2.6 e-2**	**6.5 e-3**	**6.2e-5**	**2.0 e-2**	**6.4 e-3**	**0.0013**
47	21	13.9	10.9	0.20	−9.1	6.5	0.16	−9.2 e-3	7.8 e-3	0.24	**2.4 e-2**	**7.7 e-3**	**0.0019**

Three pedigrees (7, 9, and 11) had no individuals with gene expression data. Global *p* = 0.091 for PC1, *p* < 2.2e-16 for PC2, *p* = 6.5e-12 for cytotoxic T-cell proportion, and *p* = 8.5e-9 for helper T-cell proportion. Bold indicates pedigrees with significant differences at α = 0.05/17(pedigrees) level (*p* < 0.0029)

**Table 2:** Univariate associations of covariates with PCs

Predictor variable	PC	B(SE)	T	p
Age	1	−0.20 (0.12)	−1.7	0.091
	2	0.026 (0.077)	0.33	0.74
	3	−0.15 (0.073)	−2.11	0.036
Sex	1	−2.73 (4.05)	−0.67	0.50
	2	0.52 (2.62)	0.20	0.84
	3	−0.010 (2.51)	−0.04	0.97
Blood pressure medication	1	−15.7 (6.5)	2.40	0.017
	2	2.46 (4.2)	0.58	0.56
	3	−8.87 (4.10)	−2.19	0.029
SBP	1	−0.18 (0.10)	−1.91	0.057
	2	0.033 (0.063)	0.52	0.60
	3	−0.12 (0.060)	−1.95	0.052
DBP	1	−0.33 (0.19)	−1.72	0.087
	2	−0.052 (0.13)	−0.42	0.68
	3	−0.30 (0.12)	−2.54	0.011
Hypertension	1	−12.2 (5.08)	−2.41	0.016
	2	1.93 (3.30)	0.59	0.56
	3	−5.87 (3.14)	−1.87	0.062

**Fig. 2:**
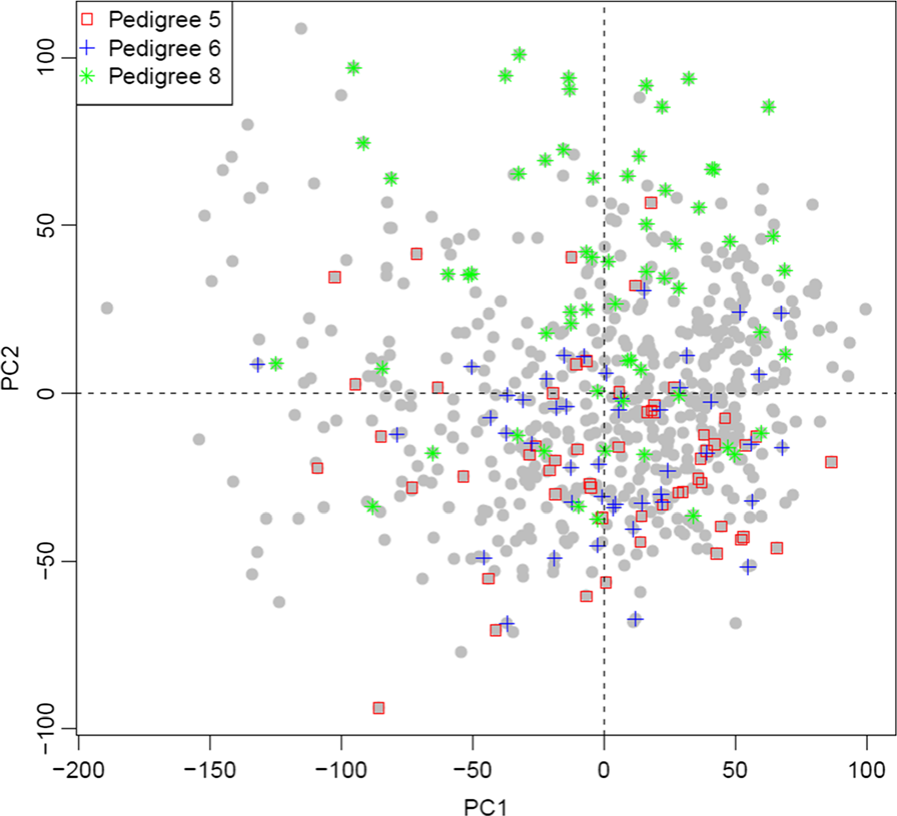
Scatterplot of PC1 vs PC2. Pedigrees 5, 6, and 8 are highlighted

**Fig. 3:**
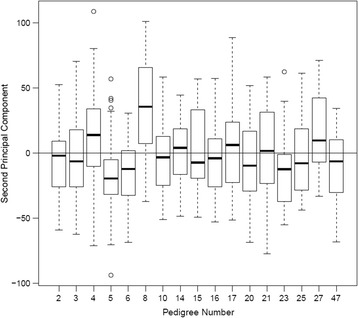
Box-and-whisker plots of the second PC for each of the individuals from the 17 pedigrees. The edges of the boxes are the upper and lower quartiles and the whiskers extend to the most extreme points within 1.5 times the interquartile range of the closest quartile. Points outside these limits are plotted individually. The location of the median in each pedigree is indicated as the thick line across the box. The horizontal line across all boxes is at 0, the overall mean of the PC

There was no significant association between pedigree number and PC1 or PC3 (*p* = 0.091 and 0.26, respectively). In contrast, there was a highly significant association between pedigree number and PC2 (*R* ^*2*^ = 0.19, *F* = 9.137, *P* < 2:2 × 10^−16^)^.^ Specifically, 3 pedigrees (5, 6, and 8) had significantly different PC2 values (see Table 1; Figs. 2 and 3). Inclusion of age, gender and the interaction between age and gender did not appreciably alter the findings, with pedigrees 5, 6, and 8 still showing significantly different PC2 values (Fig. 3). PCs were also estimated from unrelated individuals (using data available from Genetic Analysis Workshop 18), and their weights were applied to the remaining subjects, but the conclusions were not altered.

### Estimated cell proportions

**Fig. 4:**
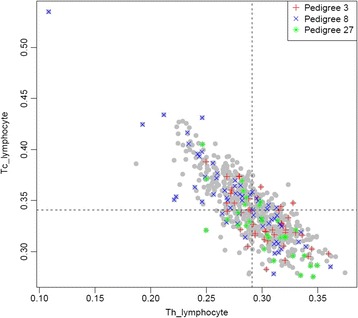
Scatterplot of estimated proportion of cytotoxic (*y-axis*) and helper (*x-axis*) T lymphocytes, with 3 outlier pedigrees highlighted

The estimated proportion of granulocytes and natural killer cells was zero for all individuals. The proportion of: monocytes ranged from zero to 0.0997 (mean = 0.0199, SD = 0.018); B lymphocytes ranged from 0.311 to 0.387 (mean = 0.348, SD = 0.011); Tc lymphocytes ranged from 0.275 to 0.535 (mean = 0.341, SD = 0.031); and Th lymphocytes ranged from 0.108 to 0.375 (mean = 0.291, SD = 0.030). The association of pedigree with cell proportions were all nominally significant but only the Tc and Th lymphocytes survived Bonferroni correction for 4 tests (*p* = 0.00012 and *p* = 0.00034; see Table 1). For Tc lymphocytes the significance is driven by differences between pedigrees 27 and 3 and the rest, whereas for Th lymphocytes it is driven primarily by differences between pedigrees 3, 5, 27, and 47 (see Table 1 and Fig. 4). One individual from pedigree 8 (ID# T2DG0800552) was identified to be a gross outlier based on Tc and Th proportions (Fig. 4), consistent with an acute viral infection. The estimated proportion of T cytotoxic and helper lymphocytes were significantly correlated with the first 3 PCs (Table 3), although this may be tautological, as some of the same gene expression data were used to estimate both measures. All cell counts (0.34 to 0.44) and the first 5 PCs (0.19 to 0.54) were significantly heritable (SOLAR [Sequential Oligogenic Linkage Analysis Routines] v4.1.3 for windows), with adjustment for age, sex, and their interaction (data not shown).

**Table 3:** Spearman rank correlations of estimated cell proportions with PCs

	Tc Lymphocyte	Th Lymphocyte	PC1	PC2	PC3
Tc lymphocyte	X	−0.80	−0.099	−0.29	−0.13
Th lymphocyte	<10^−16^	X	0.23	0.14	0.50
PC1	0.012	3 × 10^−9^	X	0.082	0.024
PC2	10^−13^	3 × 10^−4^	0.04		−0.014
PC3	0.0014	<10^−16^	0.54	0.72	X

Coefficients are above the diagonal, *p* values are below the diagonal

## Discussion

A large number of PCs are detected in the microarray gene expression data. Although age, sex, and other clinical factors were not associated with the 3 first PCs, pedigree number was highly significantly associated with PC2, with 3 pedigrees being gross outliers. Pedigree differences in PC2 could be from genetic variation that is related to pedigree membership that is influencing gene expression. Alternatively, it could be a result of pedigree differences in technical procedures or in the proportion of different cell types in those subjected to analysis. Cell proportions were estimated and 1 individual was shown to be a gross outlier and power may be improved by exclusion of such subjects. An overlapping set of 2 and 4 pedigrees had significant differences in the estimated proportion of Tc and Th lymphocytes, respectively. The analysis did not take pedigree structure into account, potentially leading to inflated type 1 error.

In general, identification of factors that are associated with differences between individuals in functional genomics measures can potentially be used to improve the power for genetic mapping studies. Because PCs and cell proportions were shown to be significantly heritable, this could motivate mapping the loci responsible.

## Conclusions

This is not the first [9, 10], nor likely the last description of possible batch effects in functional genomic data. According to the description of the GAW19 expression data, the lab method was as described in Göring et al. [2], while the data that was distributed underwent different processing [1], mostly focused on providing data for probes where the “detection *p* value” was consistent, with detectable expression across most individuals. It is unlikely that such preprocessing would produce PCs that we observed in the data.
